# Prevalence and associated factors of unhealthy food consumption among 6–23-month-old children in South Ethiopia; A community–based cross-sectional study

**DOI:** 10.1371/journal.pgph.0005311

**Published:** 2025-10-16

**Authors:** Abraham Anbesie Sapo, Tamirat Gezahegn Guyo, Habtamu Wana, Bahiru Mulatu Kebede, Fasika Merid, Kassahun Tamene Andarige, Habtamu Samuel, Adane Alto Mengesha, Serekebrhan Sahele Salile, Temesgen Mohammed Toma

**Affiliations:** 1 Department of Environmental Health, Arba Minch College of Health Sciences, Arba Minch, Ethiopia; 2 Department of Public Health, Arba Minch College of Health Sciences, Arba Minch, Ethiopia; 3 Department of Midwifery, Arba Minch College of Health Sciences, Arba Minch, Ethiopia; 4 Department of Nursing, Arba Minch College of Health Sciences, Arba Minch, Ethiopia; 5 Public Health Emergency Management Directorate, South Ethiopia Region Health Bureau Public Health Institute, Jinka, Ethiopia; African Population and Health Research Center, KENYA

## Abstract

Unhealthy food consumption in children is an emerging public health problem and has various health effects on children. Overweight/obesity in children is increasing at an alarming rate due to an unhealthy diet and other associated factors. Hence, determining the unhealthy food consumption and responsible factors among 6–23-month-old children is vital to conducting a targeted intervention. A community-based cross-sectional study was conducted among 392 randomly selected children whose ages were between 6–23 months at Arba Minch City, Gamo Zone, from March 12, 2024, to April 30, 2024. Data were collected by face-to-face interviews using a pretested structured questionnaire. A binary logistic regression model was fitted to identify factors associated with unhealthy food consumption. In bi-variable analysis, variables with a p-value <0.25 were candidate variables for multivariable logistic regression analysis. An adjusted odds ratio with corresponding confidence interval was used to determine the strength of the association. A p-value <0.05 was used to declare statistical significance. The prevalence of unhealthy food consumption among children aged 6–23 months was 52.8% (95% CI: 47.7, 57.9). Age 12–17 months [AOR = 1.77; 95% CI = 1.77; 1.05, 2.97], bottle feeding [AOR = 2.36, 95%CI = 1.48, 3.75], sub-optimal dietary diversity score [AOR = 2.08; 95% CI = 2.08; 1.24, 3.49], no postnatal care visit [AOR = 2.39, 95% CI = 1.07, 5.33], and insufficient maternal knowledge of child-feeding[AOR = 1.65; 95% CI = 1.01, 2.70] were significantly associated with unhealthy food consumption. Over half of children aged 6–23 months consume unhealthy food in the city. Unhealthy food consumption was influenced by being at a younger age, bottle feeding history, sub-optimal dietary diversity, no postnatal care visit, and low maternal child feeding knowledge among these young children. Designing interventions aimed at boosting maternal understanding of child feeding practices and improving health care services with a focus on children's healthy diet status in the city is highly encouraged.

## Introduction

Unhealthy food is a generic term for all kinds of foods that are rich in energy, because they contain a lot of fat and sugar, as well as salt, but are relatively low in other important nutrients such as protein, fiber, vitamins, and minerals [1]. Unhealthy foods are exposed in numerous forms as junk foods, quick meals, processed foods, ready-to-eat foods, conventional foods, and canned foods. Something useless, old, and hazardous to health is classified as cheat foods under unhealthy foods [2].

There are approximately 13 million children under the age of five, accounting for 16 percent of the total population; if current trends continue, by 2050, there will be 58 million children under the age of 18, which is 6% of the African population [3]. A target population of 1.4 billion low-income people in developing countries is vulnerable to unhealthy food markets, with one in five deaths linked to unhealthy foods [4].

One in three parents of young children in Australia, Ethiopia, Ghana, India, Mexico, Nigeria, Serbia, and Sudan fed their under-two-year-old child at least one processed or ultra-processed food or drink every day [5]. Internationally frequent and very frequent fast-food consumption was reported in 4% and 13%, respectively, of children between the ages of 6 and 23 months [6]. In the 2010s, 14 countries with some of the lowest incomes in the world had newly developed a double burden of malnutrition, which is secondary to unhealthy food consumption [7]. Data from the Ethiopian Demographic Health Survey (EDHS) show that up to 32% of infants and children aged 6–23 months are fed sugary foods [4,8,9]. In Ethiopia overall, 15% of children aged 6–23 months consumed unhealthy foods. The number of young children who consume unhealthy foods in urban areas is twice the number in rural areas [10].

Unhealthy food consumption in children is an emerging public health problem and has various health effects on children. Overweight/obesity in children and adolescents is increasing at an alarming rate due to an unhealthy diet and other associated factors. About 1 in 3 children aged 6–23 months who consumed sweet beverages are overweight/obese in Sub-Saharan Africa [3,7,11]. Identified cardiovascular risk factors in overweight/obese children and adolescents are primarily metabolic syndrome, hypertension, dyslipidemia, diabetes, and glucose intolerance [7,12,13]. Unhealthy food consumption also has a tremendous effect on the economy of a country. For instance, in developed countries, the annual economic costs of unhealthy diets ranged from €1.4 billion in Australia (AU$2 billion) to €4.5 billion in China (US$4.2 billion), €8.5-9.5 billion in the United Kingdom (£5.8-6 billion), and $114.5 billion in the United States [14,15].

Even though the progress of under-nutrition status has slowed in the last two decades [16], urbanization and a high rate of population growth are the leading factors for unhealthy food consumption of children, aligning with the maternal educational and low socio-economic status of the country [17–20]. Changes in country status from low-income to upper-middle-income led to nutritional shifts resulting in the double burden of malnutrition. With the speed of change in food systems, more people are being exposed to both forms of malnutrition at different points in their lives, which further increases harmful health effects in the country [21,22].

The World Health Assembly adopted a comprehensive implementation plan on maternal, baby, and early child nutrition in 2012 to meet six global targets by 2025 and ultimately eliminate all kinds of malnutrition by 2030 [23]. Different strategies and policies were developed and implemented in Ethiopia to address nutrition-related problems, although the majority of them were nutrition-focused [24,25]. Different malnutrition management interventions have significant results; the national level of stunting has decreased, while the incidence of overweight children under the age of five has grown from 1.8% in 2011 to 2.1% in 2019 [10,26].

Arba Minch City is one of the largest cities in Southern Ethiopia and the country's tourist center, owing to its diverse natural and cultural heritages, which are popular with both foreign and domestic tourists. Due to the new regional state organization, the city is currently experiencing rapid expansion of urban and pre-urban villages. In addition to its advantage of simplicity of life, urbanization can contribute to lifestyle changes that lead to nutritional transition and the development of non-communicable diseases. Evidence is scarce on unhealthy food consumption and responsible factors among children under five years old; it is important to identify the site-specific evidence to generate substantial information. Moreover, the effect of important variables, including maternal autonomy, maternal knowledge of children's feeding practices, and children's dietary diversity scores (DDS), was not assessed by previous studies. As a result, this study aimed to determine the prevalence of unhealthy food consumption and its associated factors among children aged 6–23 months in Arba Minch City of Gamo Zone, South Ethiopia.

## Method and materials

### Study design, period, and study area

A community-based cross-sectional study was employed from March 12, 2024, to April 30, 2024. The study was carried out in Arba Minch City of Gamo Zone, South Ethiopia. Arba Minch City is one of the administrative centers of the South Ethiopia Regional State and the Gamo Zone. It is located at a distance of 435 Kilometers (KM) in the south direction of Addis Ababa, the capital city of Ethiopia. Currently, there are 8 Kebeles (Kebele is the lowest administrative entity, serving as the main point of contact for inhabitants and delivering basic services at the neighborhood or community level) in Arba Minch City. Arba Minch’s built-up area has risen by 780 km^2^ as a result of increasing urbanization. The built-up area has grown at a rate of 41% each year [27]. According to the 2022 Municipal Administration and Health Department Report, the total population is 125,562, of whom 63,032 (50.2%) are females, 19578 (15.6%) are under-five children, and 6,504 (5.18%) are under two years old children. Concerning urbanization and tourism, the spread of supermarkets and stores for various packaged foods has increased in the last few decades, yet the city is known for its fruit and vegetable production [28].

### Population

All infants and young children aged 6–23 months residing in Arba Minch City with their mothers or caretakers for at least 6 months were the source population. Randomly selected infants and young children aged 6–23 months residing in Arba Minch City for at least six months with their mothers or caregivers during the study period were the study population. Pairs of all mothers/caregivers and their children aged 6–23 months residing in Arba Minch City for at least six months before the study period were included. Mothers/caregivers of children aged 6 – 23 months who are seriously ill/mentally impaired and unable to provide information/make an interview were excluded from the study.

### Sample size determination and sampling procedure

The sample size was determined using a single population proportion formula by considering the following assumptions: 95% two-sided level of confidence, 5% margin of error, and 63.7% proportion of unhealthy food consumption from the prior study conducted in Gondar [17].


$$\begin{array}{c} n=\frac{{Z\alpha }^{\text{2}}\;p(\text{1}-p)}{d^{\text{2}}};\text{ n}=\frac{{\left(\text{1}.\text{9}\text{6}\right)}^{\text{2}}\;*\;\text{0}.\text{6}\text{3}\text{7}(\text{0}.\text{3}\text{6}\text{3})}{{\text{0}.\text{0}\text{5}}^{\text{2}}}\;,\text{ n}=\text{ 356} \end{array}$$

After considering the 10% non-response rate, the sample size for this objective was 392.

**Fig 1:**
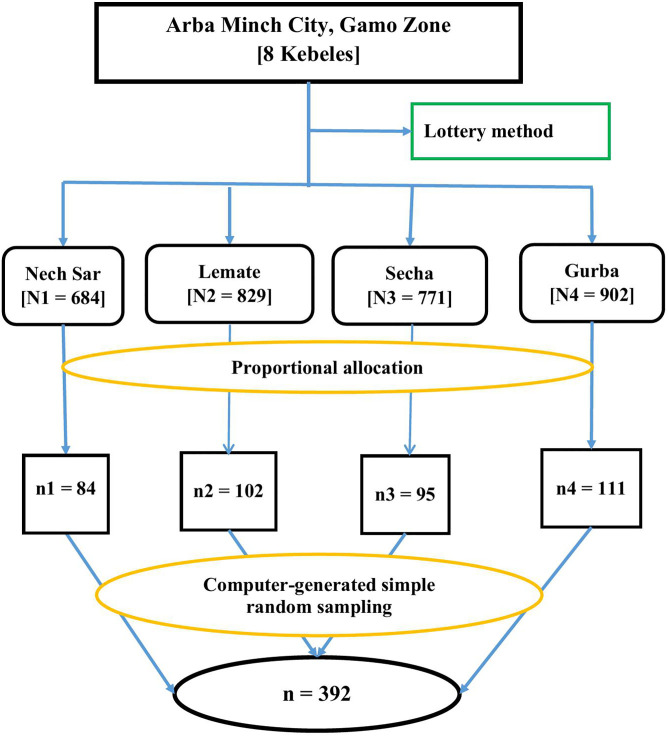
Schematic presentation for selecting participants for a study assessing unhealthy food consumption and associated factors among 6-23 months old children in Arba Minch City, South Ethiopia, 2024.

To select study participants, a simple random sampling technique was employed. Of eleven Kebeles (Nech Sar, Sekela, Lemat, Secha, Bere, Gurba, Chano Shara, and Bola Gurba) found in Arba Minch City, four Kebeles were chosen using a lottery method. The Kebeles were chosen using a lottery method due to their homogeneity in socio-demographic, cultural, and living styles within a single city. This was followed by a computer-generated simple random sampling of study subjects (children aged 6–23 months) from the four selected Kebeles. For this purpose, the number of children aged 6–23 months was obtained from the selected Kebeles’ health post family folder. The number of children (6–23 months) included in the study from each Kebele was decided using proportional allocation to size. Then, a sampling frame of children aged 6–23 months was prepared for each selected Kebele, and it was entered into Emergency Nutrition Assessment software (ENA for SMART) for random selection of 392 subjects (Fig 1).

### Data collection instrument, personnel, and procedure

Data were collected using a pretested and structured questionnaire to collect necessary information. Unhealthy food consumption status was measured using the World Health Organization's (WHO) Infant and Young Child Feeding (IYCF) indicators and reviewing relevant literature [5,12,17,29–31]. According to the WHO indicator, unhealthy food consumption was measured as a percentage of children 6–23 months of age who consumed selected sentinel unhealthy foods during the previous day. This sentinel unhealthy food contains four categories of unhealthy foods. Category one: Candies, chocolate, and other sugar confections, including those made with real fruit or vegetables like candied fruit or fruit roll-ups; Category two: frozen treats like ice cream, gelato, sherbet, sorbet, popsicles or similar confections; Category three: cakes, pastries, sweet biscuits, and other baked or fried confections that have at least a partial base of refined grain, including those made with real fruit or vegetables or nuts, like apple cake or cherry pie; and category four: chips, crisps, cheese puffs, French fries, fried dough, instant noodles, and similar items that contain mainly fat and carbohydrate and have at least a partial base of a refined grain or tuber. These foods are also often high in sodium [8].

The questionnaire was written in English first and then translated into Amharic. It includes a variety of independent variables, including socio-demographic characteristics of parents, newborns, and young children, household-related factors, and maternal health services-related factors. Unhealthy food consumption status was measured using a single 24-hour recall method. Data was collected using eight BSc Nurses and four public health officers who supervised the overall data collection process and managed the data collection procedure. Data were collected using a face-to-face interview.

### Variables and measurement

The dependent variable was unhealthy food consumption, which was dichotomized as Yes/No. The independent variables include; Socio-demographic and economic-related variables: religious status of the caregiver, caregiver's relation to a child, age of the caregiver, marital status of caregiver, education level of caregiver, occupation of caregiver, education level of father, family size, and wealth index; Child-related factors: age, sex, breastfeeding status, birth order, bottle feeding experience, initiation of complementary feeding and growth monitoring program (GMP) service, and dietary diversity score (DDS); Maternal-related factors: antenatal care (ANC) follow-up history, place of delivery, postnatal visit history, maternal autonomy, and maternal knowledge of feeding practice.

**Unhealthy food:** To determine each kid's unhealthy food consumption (UFC), the mother will be asked to identify all foods consumed by the child in the 24 hours before the survey, such as juices, soda, sugary coffee or tea, candy, chocolate, cakes, sweet biscuits, ice cream, potato chips, and instant noodles. Children who consumed at least one food from the above lists were judged to be consuming unhealthy foods; otherwise, they were regarded to be consuming healthy foods [8].

**Postnatal care visit:** According to the WHO 2022 recommendation, PNC has four contacts; the first within 24 hours of delivery, the second contact between 48–72 hours, the third contact between days 7–14, and the fourth contact at six weeks after birth. Information about breastfeeding was recommended to be provided at every visit. The mother and baby were indicated as having received PNC if they had one or more visits [32].

**Growth monitoring and promotion:** GMP utilization was assessed by collecting a child's GMP and requiring them to participate in GMP at least once in the previous three months [17].

**Wealth index:** The household wealth index was determined using principal component analysis (PCA) by considering household assets, such as house, livestock, and agricultural land ownership. First, variables were coded between 0 and 1. After checking the assumptions, the wealth index was analyzed using PCA, and finally, the factor scores were summed and ranked into poor, medium, and rich wealth indexes [17,33].

**Maternal knowledge of child feeding:** Was assessed by twelve-item questions with “yes” or “no” responses. A score of 1 was given for the correct and 0 for the incorrect response, and caregivers/mothers who scored above the mean or median were categorized as having “insufficient knowledge,” while those who scored the mean and fewer were categorized as having “sufficient knowledge [34,35].

**Child dietary diversity score**: it was assessed and categorized as sub-optimal if < 4 and optimal if ≥ 4 food categories were consumed per every meal [36].

**Maternal autonomy**: It was measured by four composite variables adapted from the 2016 Ethiopia Demographic Health Survey Tool. The first three questions were related to ‘mobility, the next three questions were related to ‘mother involvement in decision-making regarding her child, the third group of questions related to ‘financial autonomy, and also, a single item on the autonomy of family planning service utilization was also asked. Mothers who had a sum value less than the median score value were categorized as having low maternal autonomy, whereas those who scored the median and above were categorized as having high maternal autonomy [37].

### Data quality management

Data quality was ensured by adopting validated tools by reviewing the literature, giving training to data collectors and supervisors, conducting pretests, and language translation. Accordingly, a two-day training was given to the data collectors and supervisors before data collection by investigators on the objective of the study, the method of data collection, and ethical issues. A pre-test was done on 5% of the sample size in Sawla City, Goffa Zone, to check the clarity and consistency of the questionnaires and checklist before the actual data collection. The pre-test was carried out to verify the consistency of the data collection tool and to improve it. Discussion on the result of the pre-test and relevant amendments was made on the tool as required. Questionnaires were primarily prepared in English and then translated into the Amharic language and back-translated to English to guarantee consistency. Each completed questionnaire was checked for completeness, clarity, and consistency at the site of data collection by the supervisors to take corrective measures. The overall activities were also monitored by investigators.

### Data processing and analysis

Data were checked, coded, cleaned, and entered into Epi-Data 3.1 and then exported to STATA version 15 for further data management and analysis. Data exploration was carried out. Descriptive statistics were computed to describe the variables in the study. After checking the assumptions, the wealth index was computed using PCA. The assumptions include: the Kaiser-Meyer-Olkin measure of sampling adequacy, Bartlett test of sphericity, eigenvalues, and communalities. Binary logistic regression analysis was fitted to identify factors associated with unhealthy food consumption. All variables with a p-value less than 0.25 in the bi-variable analysis were selected as candidate variables for the multivariable logistic regression analysis. A multivariable logistic regression analysis was performed using a backward likelihood ratio method for the candidate variables to identify independent factors associated with unhealthy food consumption. Adjusted odds ratio (AOR) with corresponding CI was used to determine the strength of the association. A p-value <0.05 was used to declare statistical significance in the final model. Multicollinearity between independent variables was checked using the variance inflation factor (VIF) value, and tolerance, with a mean VIF = 2.14 showed no threat of multicollinearity. Model fitness was checked using the Hosmer-Lemeshow goodness-of-fit test (P-value = 0.31). Finally, the findings of the study were presented using texts, tables, and figures.

### Ethical consideration

Before the study began, ethical clearance was sought from the Institutional Review Board (IRB) of Arba Minch College of Health Sciences with a reference number of AMCHS/01/20/3310. Support letters were also obtained from the research and community service directorate office. An official permission letter was obtained from the Arba Minch City Health Office. Written informed consent was obtained from the mother/caregiver ≥18 years old (assent form for mother/caregiver<18 years old) of each child. Also, informed written consent was obtained from the legal guardian of the mother/caregiver<18 years old. Confidentiality of information was assured throughout the study process. For participants found to have unhealthy food consumption, counseling on healthy diet consumption was provided, and advised to undergo further evaluation of nutritional status.

## Results

### Socio-demographic and economic characteristics

This study included 390 caregiver pairs and 6- to 23-month-old children, with a 99% response rate. Of the youngsters who participated in the study, 227 (58%) were female, and 205 (52.6%) were between the ages of 12 and 17 months. In the study, the majority of caregivers, 375 (96.2%), were mothers. Out of the 183 (46.9%) mothers included in the study, the age range was between 25 and 30 years. Among these mothers, a significant proportion (89.7%) were married women. Additionally, in the study, 195 mothers (50%) identified themselves as Protestant followers. Regarding marital status, 350 (89.7%) were married, and a considerable number of mothers, 170 (43.6%), reported being housewives. Furthermore, 89 mothers (22.8%) held diplomas or higher educational qualifications. In terms of media exposure, a significant proportion of mothers 236 (60.5%) reported having no exposure to media. Regarding the household's fathers, 117 (30%) were government workers, while 151 (38.7%) held diplomas or higher qualifications (Table 1).

**Table 1:** Socio-demographic and economic characteristics of mothers/caregivers and children aged 6–23 months at Arba Minch City, South Ethiopia, 2024 (N = 390).

Variable	Frequency	Percent
Sex		
Male	163	41.8
Female	227	58.2
Number of children under 5 years		
One	259	66.4
Two	126	32.3
Three and above	5	1.3
Age of the child		
6–11 months	105	26.9
12–17 months	205	52.6
18–23 months	80	20.5
Age of the mother (in years)		
18-24	91	23.3
25-30	183	46.9
31-35	94	24.1
≥ 36	22	5.6
Religion		
Orthodox	159	40.8
Protestant	195	50.0
Muslim	29	7.4
Catholic	7	1.8
Marital status		
Married	350	89.7
Divorced	16	4.1
Widowed	10	2.6
Unmarried/Single	13	3.3
Occupation of the mother		
Housewife	170	43.6
Merchant	62	15.9
Government	61	15.6
Private	53	13.6
NGO	6	1.5
Daily labor	28	7.2
Maternal educational status		
Unable to read and write	52	13.3
Able to read and write	36	9.2
Primary school	100	25.6
Secondary school	113	29.0
Diploma and above	89	22.8
Caregiver's relation to the child		
Mother	375	96.2
Grandmother	14	3.6
Other	1	.3
Media exposure of the mother		
Yes	154	39.5
No	236	60.5
Obtained infant feeding information during PNC		
Yes	334	85.6
No	56	14.4
Educational status of the Father		
Unable to read and write	18	4.6
Able to read and write	34	8.7
Primary school	73	18.7
Secondary school	114	29.2
Diploma and above	151	38.7
Father's Occupation		
Merchant	73	18.7
Private	121	31
Government	117	30
NGO	17	4.35
Farmer	6	1.53
Daily labor	56	14.35
Wealth index		
Poor	144	36.9
Middle	116	29.7
Rich	130	33.3

### Maternal and healthcare-related characteristics

A high percentage of 381 (97.7%) of the participating mothers had received antenatal care (ANC) visits. Additionally, the majority of mothers, 376 (96.4%), gave birth in a health institution, highlighting the importance of professional care during childbirth. Moreover, among the mothers who took part in the study, 355 (91%) had postnatal care (PNC) visits; of those who had PNC visits, 334(85.6%) got health education about infant feeding during these visits. Among mothers/caregivers who participated in the current study, 151(38.7%) had insufficient knowledge regarding child-feeding practices (Table 2).

**Table 2:** Maternal and health-care-related characteristics among children aged 6-23 months in Arba Minch City, South Ethiopia, 2024 (N = 390).

Variables	Frequency	Percent
ANC contact during the recent birth		
Yes	381	97.7
No	8	2.1
Place of birth		
Home	14	3.6
Health center	376	96.4
PNC visit		
Yes	355	91.0
No	35	9.0
Obtained infant feeding information during PNC		
Yes	334	85.6
No	56	14.4
Maternal knowledge of child feeding practices		
Insufficient	151	38.7
Sufficient	239	61.3

### Children and healthcare-related characteristics

Children who begin supplementary feeding at the age of six months account for 152 (39%), while those who use GMP services account for 352 (90.3%). The current study identified that among children who participated in the study, 333 (85.4%) breastfed, 290 (74.4%) had a history of exclusive breastfeeding, and 252 (64.6%) had experience with bottle feeding, respectively. Of the children who participated in the current study, 56(14.44%) had sub-optimal dietary diversity scores (Table 3).

**Table 3:** Child and health-care-related characteristics among children aged 6-23 months in Arba Minch City, South Ethiopia, 2024 (N = 390).

Variable	Frequency	Percent
Complementary feeding initiation age of the child		
< 6 months	89	22.8
At 6 months	152	39.0
> 6 months	149	38.2
GMP service utilization		
Yes	352	90.3
No	38	9.7
Current breastfeeding status		
Yes	333	85.4
No	57	14.6
Experience with bottle-feeding		
Yes	252	64.6
No	138	35.4
Exclusive breastfeeding history		
Yes	290	74.4
No	100	25.6
Dietary diversity score (DDS)		
Optimal	334	85.6
Sub-optimal	56	14.4

### Prevalence of unhealthy food consumption

**Fig 2:**
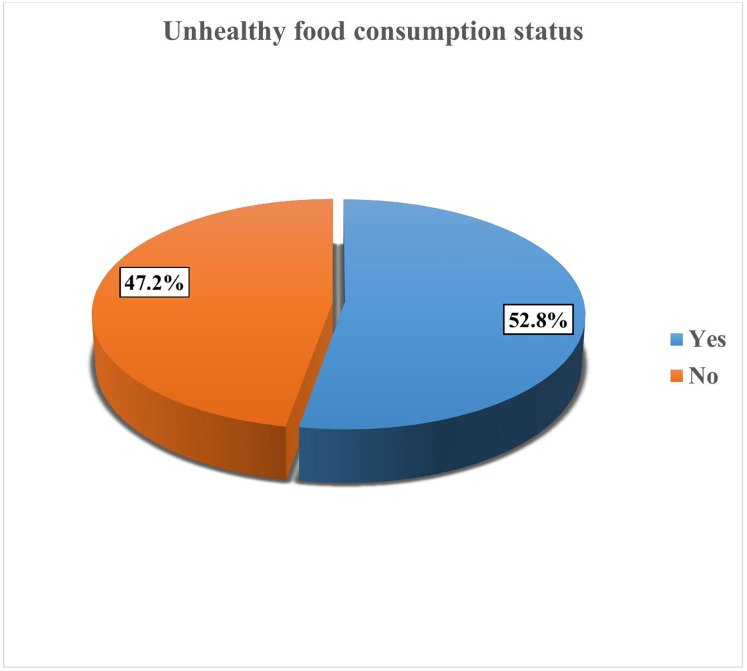
Prevalence of unhealthy food consumption status among children aged between 6-23 months in Arba Minch City, Southern Ethiopia, 2024.

In the current study, the prevalence of unhealthy food consumption among children aged 6–23 months was 52.8% (95% CI: 47.7, 57.9) (Fig 2).

### Factors associated with unhealthy food consumption

In bi-variable logistic regression, for unhealthy food consumption status among children aged 6–23 months socio-demographic characteristics, child and parental health service utilization characteristics like age of the child, sex, wealth index, PNC visits, experience with bottle-feeding, current breastfeeding status, GMP service, and media exposure were significantly associated with unhealthy food consumption on p < 0.25 and those variables were added on multivariate logistic regression. After controlling for confounding variables, in multivariable logistic regression analysis, factors such as the age of the child, postnatal care visit, experience with bottle feeding, child DDS, and maternal knowledge of child feeding practice were significantly associated with the unhealthy food consumption of the child aged between 6–23 months at p-value <0.05. Children experiencing bottle feeding were 2.36 times more likely to consume unhealthy food as compared to those with no experience of bottle feeding [AOR = 2.36, 95%CI = 1.48, 3.75]. Children aged 12–17 months were 77% high likely to consume unhealthy food than those aged 6–11 months [AOR = 1.77; 95% CI = 1.77; 1.05, 2.97]. Children who have a sub-optimal dietary diversity score were 2.08 times more likely to consume unhealthy food as compared to those who have optimal dietary diversity [AOR = 2.08; 95% CI = 2.08; 1.24, 3.49]. Mothers who have no postnatal care visits were 2.39 times more likely to provide unhealthy food than those having postnatal care visits [AOR = 2.39, 95% CI = 1.07, 5.33]. Mothers who have insufficient knowledge of child-feeding practice were 65% more likely to provide unhealthy food to children compared to those who have sufficient knowledge of child-feeding practice [AOR = 1.65; 95% CI = 1.01, 2.70] (Table 4).

**Table 4:** Bivariable and multivariable logistic regression analysis for factors associated with unhealthy food consumption among children aged 6-23 months in Arba Minch City, South Ethiopia, 2024 (N = 390).

Variables	Unhealthy food consumption		COR (95% CI)	AOR (95% CI)	P-value
	Yes N (%)	No N (%)			
Age of the child					
6–11months	48 (45.7)	57 (54.3)	1	1	
12–17 months	117 (57.1)	88 (42.9)	1.58 (0.98, 2.53)	1.77 (1.05 2.97)	0.032*
18–23 months	41 (51.2)	39 (48.8)	1.23 (0.70, 2.24)	1.05 (0.56, 1.96)	0.88
Sex					
Male	97(24.9)	70(17.9)	1	1	
Female	109(27.9)	114(29.3)	0.78 (0.52, 1.17)	0.64 (0.41, 1.00)	0.051
Household wealth index					
Poor	85(23.6)	59(15.1)	1.36 (0.84, 2.19)	1.16 (0.69, 1.97)	0.57
Middle	54(13.8)	62(15.9)	0.82 (0.50, 1.35)	0.67 (0.38, 1.16)	0.15
Rich	67(15.4)	63(16.2)	1	1	
Postnatal care visits					
No	2(0.5)	6(1.5)	2.07 (0.98, 4.36)	2.39 (1.07, 5.33)	0.034*
Yes	204(52.3)	178(45.6)	1	1	
Age at initiation of complementary feeding					
< 6 months	40(10.3)	49(12.6)	0.93 (0.55, 1.58)	0.71 (0.40, 1.27)	0.25
At 6 months	71(18.2)	81(20.8)	1	1	
> 6 months	95(24.4)	54(13.8)	2.01 (1.27, 3.18)	1.53 (0.90, 2.60)	0.11
Experience with bottle-feeding					
Yes	151(38.7)	103(26.4)	2.06 (1.35, 3.13)	2.36 (1.48, 3.75)	<0.001*
No	55(14.1)	81(20.8)	1		
Current breastfeeding status					
No	37(9.5)	20(5.1)	1.80 (1.00, 3.22)	1.43 (0.74, 2.77)	0.28
Yes	169(43.3)	164(42.1)	1	1	
GMP Service					
No	10(2.6)	14(3.6)	1.60 (0.80, 3.20)	1.39 (0.64, 3.01)	0.41
Yes	196(50.2)	170(43.6)	1	1	
Child dietary diversity score (DDS)					
Sub-optimal	14(3.6)	42(10.8)	1.96 (1.24, 3.08)	2.08 (1.24, 3.49)	0.006*
Optimal	192(49.2)	142(36.1)	1		
Maternal knowledge of child feeding practices					
Insufficient	95(24.3)	111(28.5)	2.03 (1.34, 3.09	1.65 (1.01, 2.70)	0.047*
Sufficient	56(14.4)	128(32.8)	1	1	
Maternal autonomy					
Low autonomy	80(20.5)	79(20.3)	0.78 (0.52, 1.16)	0.65 (0.42, 1.02)	0.063
High autonomy	129(33.1)	105(26.9)	1	1	
Media exposure					
No	128(32.8)	120(30.8)	1.43 (0.95, 2.15)	0.76 (0.48, 1.19)	0.23
Yes	78(20)	64(16.4)	1		

## Discussion

This study was done to determine the prevalence of unhealthy food consumption and associated factors among children aged 6–23 months. Accordingly, the prevalence of unhealthy food consumption was 52.8% (95% CI: 47.7%, 57.9%). Moreover, the age of the child, absence of postnatal care visits, history of bottle feeding, sub-optimal child dietary diversity, and insufficient maternal knowledge of child feeding practice were identified to be the predictors of unhealthy food consumption.

The current study reported that the prevalence of unhealthy food consumption was 52.8%. Utilization of the WHO-recommended standard tool to measure the outcome variable, unhealthy food consumption, enhanced the validity and accuracy of our findings. In addition, the data collection procedure was standardized through pre-testing and training of the data collectors and supervisors, double data entry was used, and exploratory data analysis was computed to identify potential outliers. Given the city's rapid expansion of urban and pre-urban villages, the finding was not surprising. Along with the simplicity of life it offers, urbanization can also lead to lifestyle changes that result in nutritional transitions. This finding is consistent with studies conducted in Lebanon, 48.9% [38], and Brazil, 43.1% [39]. This finding is lower than studies carried out in Nepal, 74.1% [31], Peru, 78% [40], and Gondar, 63.7% [17]. The possible explanation for this discrepancy might be due to the difference in sample size, as the studies done in Gondar [17] and Nepal [31] utilized a larger sample size. Moreover, the difference in socio-demographic characteristics and the difference in the study population may also account for the discrepancy in the study done in Nepal [31], taking children aged 12‐23 months as the study population. On the contrary, this finding is higher as compared to studies done in Iran 11.3% [41], Brazil 25.8% [42], and an overall survey in Ethiopia 15% [10]. There is similarity in study setting between the current study and studies done in Nepal and Brazil, for they are all community-based and also the study from Brazil was from an urban city like the current one. In addition, the aforementioned studies utilized the 24-hour dietary recall method to assess the outcome variable and also consistent in study design. Moreover, the study population was similar for the current study and the studies conducted in Iran and Brazil. The plausible explanation for the discrepancy might be variations in a study setting as the study done in Iran is healthcare facility-based [41]. Study design and study population differences could also be the contributing reasons since the study done in Ethiopia is a descriptive survey and recruited all under-five children [10]. Moreover, the difference in sample size and socio-cultural characteristics might also be plausible explanations for the differences.

In the current study, children aged 12–17 months were 77% more likely to consume unhealthy food than those aged 6–11 months. This is supported by the findings from a study conducted in Brazil [42], which reported that older children were at increased risk of consuming unhealthy food when compared to those aged 6–11 months. It was also consistent with the findings from studies conducted in Nepal [31] and Lebanon [38], which reported that the odds of unhealthy food consumption increase as the age of the child increases. Moreover, this finding is supported by a study done in Indonesia, which reported that the odds of unhealthy food consumption among children aged 12–17 months were more than five-fold times higher than those aged 6–11months [43]. On the contrary, a study from Gondar, Ethiopia, showed that children aged 6–11 months had a 47% higher likelihood of unhealthy food consumption as compared to those aged 18–23 months [17]. The possible explanation for this might be socio-cultural variation, study setting, and early engagement in family food as the age increases in Gondar. Whereas, in this study setting, three-fourths of children started complementary feeding at six months and above, which may have less chance of engaging in unhealthy food. Furthermore, it might be because some caregivers are choosing to feed their children snacks on processed commercial products. Reduction of unhealthy food consumption by children aged 6–23 months can be attained by improving maternal or caregiver knowledge and practice on the preparation of complementary feeding.

Children who had a history or experience of bottle feeding were at more than two-fold risk of consuming healthy food than their counterparts. The current study also showed that more than 60% of the children had a history of bottle feeding among which about 60% experienced unhealthy food consumption. A possible explanation is that in this study, one-fourth of the children begin complementary feeding at the age of less than six months, and one-fourth of the children had no history of solely breastfeeding, which provides a foundation for food consumption using a bottle. Moreover, about 57% of moms in this study are not housewives, which creates opportunities for commercial food consumption for the child. Furthermore, children who are bottle-fed might be introduced to solid foods, including unhealthy options like processed snacks and sugary drinks, at an earlier age than breastfed children. This finding implies that healthy child-feeding practices can be improved by stopping bottle feeding or using it with greater sanitary precautions, and using it for either breast milk or other recommended complementary food by boiling.

Children who have a sub-optimal dietary diversity were nearly two-fold times likely to consume unhealthy food as compared to those who have an optimal dietary diversity score. This is likely due to the following factor in the study setting: insufficient understanding of newborn feeding practice could be a significant problem. Low socioeconomic status may also play a role. Families with financial constraints may have limited means to acquire a wide range of nutritious foods. The media has the potential to significantly influence dietary choices and preferences. However, low media exposure in this research context may also have an impact on the dietary diversity of the children. Furthermore, current market inflation at the country level may exacerbate the problem. Rising food prices and restricted availability of affordable, nutritious options may further limit dietary diversity, resulting in higher consumption of unhealthy foods. This finding emphasizes the necessity of enhancing the implementation of policies and initiatives aimed at reducing the consumption of unhealthy foods by promoting a more diversified diet, which would assist the city in its efforts to improve child health.

The current study revealed that mothers who did not have postnatal care visits were more than two times more likely to provide unhealthy diets than those who had postnatal care visits. The plausible explanation for this is that a mother who has not had a postnatal care visit should not opt for knowledge regarding infant and young child feeding practices, which limits her ability to select and prepare food for her small newborn and children. In this study, minimal media exposure and low maternal education will be the contributors to this. In addition, postnatal care visits for mothers reduce unhealthy food consumption in children due to health facilities counseling and advice on healthy feeding habits. Postnatal care visits for mothers reduce unhealthy food consumption in children due to health facilities counseling and advice on healthy feeding habits.

Mothers who have insufficient knowledge of child-feeding practices were 65% more likely to provide unhealthy food to children compared to their counterparts. More than half of the mothers of the children had insufficient knowledge of child-feeding practices. This might be because mothers who were knowledgeable about infant and young child feeding recommendations can feed their children healthy food, and the reverse for those with insufficient knowledge [44]. Limited educational status may favor limited knowledge regarding infant feeding practice, which is substantiated by a study conducted in Brazil [39] and Gondar [17]. The low educational status of the mother is closely related to unhealthy food consumption of children aged 6–23 months. As a result, one possible reason for this finding is that in this study setting, one-fourth of the mothers do not have a formal educational status. In addition, minimal media exposure limits access to information and the development of knowledge on child feeding. This finding implies that improving maternal knowledge of child-feeding practices can minimize the increased unhealthy food consumption practices in the city.

The current study finding implies that a significant proportion of children are consuming unhealthy food. Hence, improving the dissemination of healthcare information about child feeding practices during prenatal and postnatal care, and promoting healthier eating practices, greatly aided by increased media attention, is crucial to lowering the prevalence of unhealthy food consumption.

### Strengths and limitations of the study

The study's strengths included being the first to examine the unhealthy food consumption status of children aged 6–23 months in South Ethiopia. In addition, it incorporated child DDS and maternal knowledge regarding child feeding practices. The 24-hour recall method was selected for this research in low-resource settings because it is simple and practical compared to a food frequency questionnaire (FFQ). This approach effectively captures the recent dietary intake of children, offering contextual information that may be missed in broader frequency assessments, particularly when examining specific events or occasions. Despite the aforementioned strengths, the study has some limitations. Using a 24-hour dietary recall is challenging in recalling portion size, and it can't accurately represent a person's typical dietary intake, as it doesn't account for day-to-day fluctuation in food choice. It is a snapshot of one day's intake and may not be representative of long-term dietary habits.

### Policy and program implication

By considering the shifting of infant and young child dietary patterns towards higher intakes of refined carbohydrates, unhealthy fats, added sugars, and salt in low-and middle-income countries initiated the WHO to add unhealthy food consumption in to IYCF indicators to prevention childhood overweight/obesity and non-communicable diseases [8]. Despite this recommendation, the current study indicated that the prevalence of unhealthy food consumption among children aged 6–23 months was high due to different predicting factors. The findings of the study are important for program, policy, and practice in the city, align with the sustainable development goal, particularly the second goal, which focuses on ending all forms of childhood malnutrition [45]. Hence, the current finding highlights that programmers and policymakers should emphasize the reduction of unhealthy food consumption for attaining SDG 2 through the comprehensive implementation of nutrition-specific and sensitive interventions based on the identified factors. Moreover, this significant public health concern needs consistent and integrated interventions from the city, regional health bureau, and other stakeholders to end all forms of malnutrition. Likewise, designing health policies and strategies should be considered to increase maternal knowledge on child feeding practice, ensure diversified diet consumption among infants and young children, and scale-up of postnatal care follow-up, which are contributors to unhealthy food consumption to enhance balanced nutritional status of the children. Furthermore, age-specific interventions and stopping bottle feeding are essential for tackling this critical public health problem.

## Conclusion

Unhealthy food consumption is a significant public health problem for children under the age of two years in the study setting. It is significantly influenced by maternal knowledge of infant feeding practices, postnatal care visits, age of the young children, child's history of bottle feeding, and sub-optimal dietary diversity score. Therefore, due emphasis should be given to providing care and information on child feeding knowledge and practice in various sessions such as family planning, immunization, and other routine health care delivery activities. In addition, designing interventions targeted at boosting maternal understanding of child feeding practices and improving health care services with a focus on children's healthy diet status in the city is highly encouraged. Moreover, a longitudinal study to determine habitual and pattern-related factors and or a qualitative study to explore parental behavioral factors was highly encouraged for future research.

## Supporting information

S1 Dataset The dataset supports the findings of the study. (XLS)
